# Dual cognitive pathways to voice quality: Frequent voicers improvise, infrequent voicers elaborate

**DOI:** 10.1371/journal.pone.0212608

**Published:** 2019-02-27

**Authors:** Inge Wolsink, Deanne N. Den Hartog, Frank D. Belschak, Ilja G. Sligte

**Affiliations:** 1 Department of Social Psychology, Faculty of Social and Behavioural Sciences, University of Amsterdam, Amsterdam, The Netherlands; 2 Department of Leadership and Management, Faculty of Economics and Business, University of Amsterdam, Amsterdam, The Netherlands; 3 Department of Brain and Cognition, Faculty of Social and Behavioural Sciences, University of Amsterdam, Amsterdam, The Netherlands; Middlesex University, UNITED KINGDOM

## Abstract

We investigate the involvement of Working Memory Capacity (WMC, the cognitive resource necessary for controlled elaborate thinking) in voice behavior (speaking up with suggestions, problems, and opinions to change the organization). While scholars assume voice requires elaborate thinking, some empirical evidence suggests voice might be more automatic. To explain this discrepancy, we distinguish between voice quantity (frequency of voice) and voice quality (novelty and value of voiced information) and propose that WMC is important for voice quality, but less for voice quantity. Furthermore, we propose that frequent voicers rely less on WMC to reach high voice quality than people who voice rarely. To test our ideas, we conducted three studies: a between-participant lab-study, a within-participant experiment, and a multi-source field-study. All studies supported our expectation that voice quantity is unrelated to WMC, and that voice quality is positively related to WMC, but only for those who rarely voice. This indicates that the decision to voice (quantity) might be more automatic and intuitive than often assumed, whereas its value to the organization (quality), relies more on the degree of cognitive elaboration of the voicer. It also suggests that frequent and infrequent voicers use distinct cognitive pathways to voice high-quality information: frequent voicers improvise, while infrequent voicers elaborate.

## Introduction

We all know people in our work context who speak up frequently, fast, and fluently and individuals who are vocally more frugal. But which of the two speaks up with the best ideas, and which cognitive processes underlie whether a high-quality message is communicated? This paper aims to explore how much cognitive elaboration employees need for communicating high-quality voice, and whether the frequent and the frugal voicer reach high quality voice through different cognitive pathways.

Voice is defined as speaking up through the constructive communication of problems, challenging opinions, and suggestions for improvement [1,2]. Voice is proactive, discretionary, planned behavior, with the intent to change something in the organization. Voice is important for organizations as management is often too far removed from the workplace to be able to oversee all haphazard, errors, inefficiencies, and improvement opportunities. Managers thus need their employees to voice in order to be aware of what needs to be changed [2]. Voicing often results in positive organizational outcomes. Whether outcomes are indeed favorable, however, likely also depends on the quality of what is voiced. We define voice quality as the novelty and value of a voiced message. Understanding how employees reach voice quality, can help making voice more effective and valuable to organizations.

Although voice research has recently recognized the importance of studying the content of voice [3] voice quality has received little research attention to date, but see [4] for an exception. In order to study voice quality, we first need to distinguish the quality of voiced content from mere voice actions (voice quantity). We thus define voice quantity as either one or more acts of voice, independent of voice content. While we know a lot about antecedents and consequences of voice quantity [5], we know only little about how a high-quality voice message is evaluated and selected in the mind, and eventually communicated to others. Our first goal is therefore to introduce the concept of voice quality to the voice discussion and, although we expect the two to be related, distinguish voice quality from voice quantity. The second goal of the present paper is to explore how voice is executed in employees’ minds, by analyzing the interplay between the quantity of voice actions and the quality of voice from a dual process theory of mind perspective.

The central principle of all dual process theories of the mind, is that there are two modes of cognitive processing, one automatic, and one controlled [6]. Dual process theories [7–10] propose that perception and behavior result of an interplay between these two cognitive systems. The first (system 1) is fast, automatic, intuitive, and heuristic, the second (system 2) is slow, deliberate, elaborate, and systematic. Dual process theories are widely applied in a variety of fields. For example, situational factors influence the likelihood we use system 1 or system 2 thinking [11] or emotion-regulation [12], and gender and personality partly influence our preferred type of cognitive processing [13,14]. In social contexts, system 1 versus system 2 processing influences how we perceive other people [15–18], how we distribute resources across groups [19], and whether we make honest or altruistic choices [20–22]. Dual process theories are thus extremely relevant for understanding cognition and behavior in the social context, and are therefore an important framework to apply to voice research.

System 2, elaborate and systematic cognitive processing is limited by Working Memory Capacity [23]. Working Memory Capacity (WMC) refers to the amount of information one can actively maintain in the attentional field and use for thoughts or actions [24]. Working memory represents those thoughts that we are aware of [25], and the Capacity of Working Memory supports conscious control over our thoughts, feelings, and actions [26]. Both individual differences in WMC as well as manipulations of WMC by means of distraction of conscious attention, have been used in previous research to study the role of elaborate thought and controlled attention in idea generation [27]. Studying how WMC relates to the quantity and quality of voice, will thus help us to understand how much elaborate conscious cognition is needed for voice behavior.

Conflicting ideas about the role of conscious, elaborate cognition in voice behavior have been presented in the literature. On the one hand, scholars propose that the challenging nature of voice augments the need for system 2, elaborate cognitive processing [28,29]. On the other hand, researchers argue that voice decisions may depend on automatic responses and schemas that operate below the level of conscious, rational decision making [2], thus indicating that voice relies more on system 1 processing. In support of the latter view, empirical evidence shows that cognitive ability is unrelated to the quantity of voice: more cognitive resources do not make employees voice more [30]. Here, we propose a way to reconcile both views, by arguing that cognitive elaboration is more important for the quality aspect of voice, than for the quantity.

Looking closely at the arguments of authors in favor of an elaborate processing view of voice [11,12], it becomes clear that the reasons why elaborate thinking is necessary for voice pertain to innovativeness of the content of voice, whereas arguments in favor of a more automatic processing view of voice pertain more to why and how people decide whether they want to voice at all, thus to the voice act, not the content. In our opinion, both views are thus correct. Which one is correct, however, depends on what voice aspect we focus on: quality, or quantity. While the mere decision to voice may be taken more or less automatically, cognitive elaboration may be important to select and communicate messages that add originality and value to the organization. Studying these aspects together but independently in relation to WMC, is thus necessary to unravel whether indeed, elaborate cognition may be less important for the quantity of voice, than for the quality of voice.

Next to its potential value to organizational practice, voice quality is thus also an important aspect of voice behavior in the area of cognitive research that has not been studied before. We present 3 studies using different methods to investigate how voice quantity and quality are related, and how they relate to individual differences in WMC and are causally affected by WMC load-manipulations. Specifically, Study 1 (lab study, between-person individual differences) and Study 2 (experiment, within-person situational differences) were designed to test the involvement of WMC in objective, behavioral measures of quantity and quality of voice. In Study 3 (multi-source survey field study), we investigated voice quantity and quality towards both colleagues and supervisors to replicate the lab-results in an organizational sample.

### Theoretical framework

To express voice is to challenge the status quo with the intent of improving the situation. Employee voice is an informal, voluntary way of constructively communicating ideas, suggestions, concerns, information about problems or opinions on work-related issues. Voice is proactive and constructive in the sense that employees who voice try to anticipate future problems in the organization as opposed to merely criticizing the current state of affairs [5]. Scholars have argued that this type of dissenting behavior helps organizations to learn from their mistakes [31] to avoid crises like the explosions of NASA-space shuttles ‘the Challenger’ and ‘the Columbia’ by breaking with [32] and to increase team-innovation and effectiveness [33].

However, voice is not always appreciated by the individuals that the ‘voicer’ attempts to influence. For example, research shows that employees who voice do not always get favorable performance evaluations [34]. Evaluations by others play a crucial role because voice is inherently social. It always involves a target. Employees can voice towards different targets including colleagues (speaking out) or supervisors (speaking up), depending upon whom they feel might be able to set the desired change in motion [35]. However, even though voice behaviors are improvement- and socially oriented [1], the challenging nature of voice makes it interpersonally risky [36]. Since voice entails social risk and is not always effective in initiating change [37], one might assume that people think carefully before they speak out or up.

#### Voice quantity

Although it seems logical that people ‘think’ before (or while) they voice, it is not that clear yet how the voice decision process develops in the mind. Until recently, the predominant focus in the empirical voice literature has been on situational and personality factors that influence the act of voicing. Early voice measures tended to focus on whether or not people voice ideas, problems, or suggestions in organizations (e.g., ‘this employee communicates his / her opinion … even if others disagree’, [1] We refer to this as voice quantity since it assesses whether or not people speak up. More recent measures of voice do include more aspects pertaining to the content of voice, yet use items that possibly combine both quality and quantity thus making it difficult to disentangle the two [29,38]. Studies show several contextual and trait variables to be related to voice, such as safety and self-esteem [39,40], leadership [35,41], personality [4], and job autonomy [42]. Cognitive and experimental research on voice, however, is scarce and insight into the cognitive processes underlying voice decisions is limited to date.

Several theoretical ideas exist on the involvement of elaborate cognition in the voice process. For example, some authors assume that voice requires elaborate cognitive processing, because it is intentional, planned, and future focused [28,29]. Others argue that voice requires a risk-reward calculus: when judgements of efficacy and safety increase, people voice; when they decrease, voice involves risk, and thus people remain silent. Factors that influence evaluations of risk and efficacy include voice climate in groups, felt psychological safety and the perceived probability of success, organizational support, and trusting relationships with voice targets [43–45]. However, even though there are many elements to consider when deciding to voice (‘how to voice, what to voice, will someone punish me, am I able to get the message across?’), that does not necessarily mean that everyone always elaborately and consciously considers all those elements. Even complex decisions such as buying a house presumably depend largely on automatic processing [46]. So even though decisions seem highly complex, they are often taken based on automated social scripts [47].

Extensive research has shown that risky decisions are often based on heuristics and biases, reflecting automatic cognitive processing [48]. Studies show that people judge whether they feel safe or at risk based on affective properties [49], which forms a much more efficient way to navigate in a complex, uncertain world than elaborate processing. People often remain unaware of their motivations for engaging in or refraining from risk taking [50–52]. Voice research indeed shows that implicit theories about the effectiveness of voice predict whether people withhold voice that might be useful for management [53–55]. Similarly, other authors suggest that decisions to voice ‘depend on general as opposed to specific effortful cognitive appraisals of context’ [56] and might ‘operate below the level of conscious, rational decision making’ [2].

When people voice frequently, it would not be efficient to use elaborative information processing every time one decides to voice. Individuals likely simply choose to engage in the behavior that felt good in the past. Speaking up or staying silent may thus be the result of learned scripts for decisions under risk, which often involve little elaborate cognitive processing. In line with this, the only study that we know of that investigated the relationship between more general cognitive resources (GMA) and voice [30] found no association. This suggests that an ability to engage in complex thinking does not influence whether people engage in voice or not. Thus, we propose that voice decisions (voice quantity) are not the result of careful cognitive elaboration, but are rather made using fast and automatic cognitive processes.

#### Voice quality

A more likely part of the voice process to involve elaborate cognitive processing, is the quality of voiced content. Although pioneering voice research has focused primarily on voice quantity (frequency), more recently, scholars have started to discriminate between types of voice on the basis of content. For example, people who feel obligated to change things at work, voice more *suggestions* (i.e. promotive voice), people who feel safe, voice more potential *problems* (i.e. prohibitive voice) [2,3], and people who self-monitor voice less deviating opinions [57]. Yet, we do not know what makes voiced information, whether it be a suggestion, a problem, or an opinion, of actual importance to the organization. While more recent content-specific measures such as promotive voice [3], constructive voice [38] or prosocial voice [58] presumably also capture quality to a certain extent, they do not explicitly disentangle quality from quantity, which is, in our opinion, necessary for studying the role of cognitive elaboration. We expect voice quality and quantity to be distinct (quantity does not always lead to quality) but inter-related components of voice behavior (e.g., frequent voicers more often experience what is valued and thus learn to voice high quality over time).

We define voice quality as ‘the discretionary constructive communication of suggestions, problems, or opinions that add novel and useful information to the organization’. This relates to creativity, which is the generation of original yet appropriate ideas in any domain [59,60]. Thus, originality or novelty plays a role in both voice quality and creativity. One other way to conceptualize voice quality would thus be to refer to creative voice, or proactive creativity [61]. However, we refer to voice quality for the purpose of contrasting the quantity and quality aspects of voice in this paper. This means that compared to organizational creativity measures [62,63], the voice quality concept has a broader focus. For example, besides sharing solutions and suggestions, it is also about pointing out important problems, and besides sharing novel ideas or challenging opinions, voice quality should always be useful. Even though usefulness is in the definition of creativity, it is usually underrepresented in creativity measures. Furthermore, in the currently available measures of creativity in organizations, there is no clear differentiation between proactive creativity (self-initiated), and reactive creativity (a response to a request from the supervisor). Since we are interested in the quality of voice as a proactive behavior, such a distinction is important.

The same proactive–reactive distinction applies for a clear differentiation of voice quality from creativity measures that are used in experimental research. Experimental research on the influence of elaborate cognition and attention on creativity uses tasks [64–66] that are reactive in nature: people are requested to generate creative ideas. They are not assumed to initiate their own creative responses and goals, and they do not socially interact with others. Investigating more proactive forms of creativity, in this case, the quality of voice, is thus important because highly creative people may generate great ideas in experiments, but never share them in a social environment. We want to study the involvement of cognitive processes not only in the generation phase, as has been done in previous work, but also within the process of deciding what to share with other people. Voice quality is thus a proactive, extra-role behavior: it refers to a voluntarily offered opinion, idea, or solution to a self-discovered problem. Voice quality is thus more social, more proactive, and contextually broader than the creativity that is typically studied in previous research.

While creativity and voice quality are thus different, we assume that they are related because both require the generation of novel information, and are thus facilitated by elaborative cognitive processing. Research suggests that Working Memory Capacity facilitates the idea generation process [65,67]. Here, we argue that WMC is likely to influence the quality of voluntarily voiced suggestions, problems, and opinions not only because it allows for original idea generation (creativity), but also because it allows for developing, selecting, and communicating those suggestions, problems, and opinions that matter to the organization (voice quality).

#### WMC: Cognitive elaboration resource

‘We constantly overestimate the power of consciousness in making decisions, but in truth, our capacity for conscious control is limited’[30].

The capacity for conscious control is reflected in the capacity of working memory [26], which is limited by the quantity of information it can contain. Imagine being in a secluded park with 3 children. Two of them are playing in the grass, but the third one attracts your attention because she gets in trouble climbing up a tree. Before you attend to the tree-climber, you glance at the other two, remembering their position. You keep this visual-spatial information in mind when you climb up the tree to get the third one, which allows you to quickly locate and check on the others while you are in the tree. Now imagine the same scenario, but in a petting zoo with 8 children during high-season. Most people would fail to remember the location of all remaining 7 children, especially considering the distraction of animals and other kids. The number of children to be remembered (storage capacity) and the ability to rule out irrelevant children (distractor interference), varies greatly between people: there is a capacity limit to the memory-system. Working memory holds limited information ‘online’ to serve cognition [68]. How much we can keep attentively online at a time (Working Memory Capacity or WMC) ultimately defines the possible depth of our thoughts as well as our capacity for conscious control [26,69].

Since WMC is central to effortful and elaborate cognitive processing [25] and attention control [70–73], it is important for performing any task that requires focused and elaborate thinking, such as planning, reasoning, and persistent future goal pursuit. More specifically, the number of items (storage capacity) one can retain in the visual working memory is assumed to be equal to the number of interrelationships between elements that can be kept active while reasoning [74]. The storage component of (visual) working memory is thus a key limiting factor in our ability to understand abstract relationships between novel items [75,76]. This means that WMC is related to fluid intelligence, but is nevertheless a different construct [77,78]. WMC is a more basic, specific measure of elaborative processing capacity, which is not distorted by domain-specific, cultural, or knowledge-dependent skills. Visual working memory tasks are only about the number of items one can retain within the visual attention scope, not about reading ability, mathematical ability, or knowledge from long-term memory (crystallized intelligence).

Another way to measure the involvement of WMC in any behavior that specifically focusses on the part of WMC that controls attention, is to put load on working memory such that it cannot be used to execute other tasks [70]. Because WMC is limited, once it is ‘full’, it is occupied and cannot be used to cognitively elaborate on other issues. In our studies, we therefore use both visual WMC measures as well as attentional load manipulations to study the involvement of elaborative processing in the quantity and quality of voice. We thus investigate WMC’s involvement in voice both in terms of individual differences in evolutionary basic, visual WMC, and in terms of causality. As argued above, we expect that with regard to the quantity of voice, there is little elaborate cognitive processing involved, and we therefore propose the following hypothesis:

*H1—WMC is unrelated to voice quantity*.

In contrast to voice quantity, we propose that the role of WMC is more important for the quality of the voiced message. In support of a link between elaborate cognitive processing and idea development, experimental research has shown that elaborate processing is important for the generation of original ideas and the execution of musical improvisation. De Dreu and colleagues [65] showed that WMC facilitates idea generation because it allows for a persistent and perseverant focus of attention on the creative task. People high on WMC are able to stay focused on the generation process for longer periods of time and generate original ideas because they consider more possible alternatives within contextual categories.

Similarly, Benedek and colleagues [64] found effects of both WM storage capacity and WM distractor interference on creative performance, indicating that idea generation is fostered by the ability to filter out distracting information and the amount of items one can retain in working memory. If thus, elaborate processing facilitates the generation of original ideas, it may also be important for the evaluation, selection, and communication of high-quality ideas, problems, and opinions. Being able to assess whether a message contains novel information presumably requires understanding of the status quo and a thorough thought process to check whether indeed, the idea, problem, or opinion is not a mere repetition of issues that were previously raised by others.

In addition to fostering the generation and communication of original ideas, we propose that cognitive processing is likely to help people select and communicate those ideas that matter in their specific context (usefulness) [79]. describes the importance of understanding and mastering ‘the context’ in order for group improvisation to lead to innovations, instead of mere chaos. According to Sawyer, the innovation process is about selecting those ideas with potential, and making incremental changes in multiple rounds of improvisations. This incremental, implementation focused, usefulness element is important for voice because voice is social and needs to help the organization, as opposed to disrupting organizational functioning. Selection and communication of ideas, problems, and opinions that are not only original, novel, and challenging, but also useful, valuable, and implementable, requires understanding and shaping of the content. This makes the ability to keep a large quantity and variety of information active in the mind (WM storage capacity) important. Furthermore, as voice is social, the ability to filter out distracting environmental noise (WM distractor interference) while focusing attention on what to say and how to say it might be needed for selecting the best idea for communication.

The arguments above suggest that WMC is relevant to voice quality because the generation, selection, and communication of messages that are both novel and useful requires elaborate cognitive processing. We thus hypothesize that:

*H2—WMC is positively related to voice quality*.

Study 1 tests our hypotheses by investigating individual differences in WMC whereas in Study 2, we manipulated WMC through distracting people during the selection of voice-messages.

## Methods Study 1

### Ethics, design and sample

Study 1 was conducted in 2013, according to the principles expressed in the Declaration of Helsinki. Informed consent (written) was obtained from all participants. All data was examined anonymously. Participants were aware that they could terminate their participation at any time, and retract their responses within 7 days after participation. Participants were first paid, and then debriefed. The data were collected over a period of 4 weeks. The same experimenter was responsible for data collection during the full data collection process. Below, we provide a short summary of our methods, a more detailed methods section is available at dx.doi.org/10.17504/protocols.io.tubensn.

This between-subjects lab study tested whether individual differences in cognitive resources (visual WMC) predict whether people voice (voice quantity, *H1*), and whether the content of people’s voice is useful and takes new information into account (voice quality, *H2*). Participants were 72 businesses students (57,7% male, 42,3% female) in all years of enrollment with an average age of 21.07 (*SD =* .21, *range* = 18–26) and a part-time job of 11.84 hours a week on average (*SD =* 7.39). They were tested for visual WMC and worked in a dyad on a fictional business case together with a team-leader. Participants had the chance, but were not required or requested to voice opinions and suggestions (voice quantity) to solve the case. The quality of the solution reached served as a measure of voice quality.

### Procedure, tasks, and measures

#### Independent variable WMC

The independent variable WMC was assessed by means of a delayed serial recognition task [56–59]. Participants performed a series of 96 trials. On each trial, participants were presented with eight [8] randomly selected pictures, appearing sequentially in the center of a laptop screen. Each stimulus remained on screen for 250 milliseconds (ms), extending the complete trial over a period of 2000 ms. After a series of eight stimuli, the screen went blank for 1000 ms during which participants had to keep all information in memory. For each set we assessed response accuracy with a perfect WMC score of 96 [80].

Working Memory Capacity was calculated with a linear transformation of accuracy into capacity as used by Sligte and colleagues [64,65]. WMC was calculated by transforming the percentage of correct responses into a capacity estimate between 0 and 8, correcting for chance level (the percentage of correct responses if participants randomly select a response, which is 0.5 or 50%). We used the following formula:

WMC=(%correctresponses‑chancelevel)*(nitemsintask*1/1‑chancelevel)

This ultimately results in: WMC = (% correct—0.5) * (8* 0.5). Performing at chance level would thus result in a capacity estimate of 0, and perfect performance would result in a capacity of 8. The number of items participants could retain in working memory ranged from 2.33 to 6.83 (*M =* 5.31, *SD* = .89).

#### Dependent variables voice quality and quantity

Following this test, participants were introduced to their team-leader with whom they performed a filler task to get acquainted. Leaders were confederates and specifically instructed and trained for the study (namely a male and a female faculty employee who were blind to the study’s hypotheses and uninvolved in the study as authors). Gender of the leaders was counterbalanced between groups. To measure voice, we provided the team (participant and leader) with an information sharing task adapted from [81]. This task is specifically useful to measure voice and initiative behaviors in the social environment, because team-members need to voice in order to get to the best solution. We specifically created a leader-follower situation because we wanted any voice from participants to be upward, discretionary, risky, challenging and extra-role: the responsibility for the task-execution was completely in the hands of the leader, and leaders had the power to distribute payment at the end of the experiment (or so we told the participants). Participants had the chance to voice, but were never specifically requested for their opinion or help during the task.

The team-leaders’ task was to find the best new dean for the faculty. There were three candidates (A, B, and C) who all had different properties, which were selected via a pre-test of 283 students on favorable and unfavorable dean-attributes. The candidate profiles were composed in such a way that based on all available information, C would be the best candidate for the job, and A and B would both be unfavorable. However, the information that was provided to the individual team-members was (as it often is in practical situations of decision making), not identical. Based on only the *leaders’* information, B would be the best option, and based on the *participants’* information, A would be best. The team thus needed to share and integrate all information to get to the optimal decision, C (high quality).

After carefully reading all information on the sheet, the leader said: ‘based on my information here, it is quite obvious that we should go for option B. Here is my information, confirming my decision’. If, at that point, the participant had proactively tried to find the best candidate for the job, s/he would have found out that from his/her perspective, A was the best choice. If the participant noticed this and wanted to mention it, s/he needed to challenge the leader and voice another option. The participant could now choose *(1)* not to voice and comply with the leader, or *(2)* oppose the leader and suggest another option (voice). Whether participants voiced or not, was our measure of *voice quantity*. *Voice quality* was assessed by the relative quality of the option participants voiced. As explained previously, the participant could either choose the less optimal option A, thus staying with his or her previous preference, or derive a new perspective from the leaders’ information and voice option C, the best option from the combined viewpoints. The outcome difference between these suggestions, is assumed to be the result of the evaluation and selection process for the voice message that integrates novel information, and is most valuable to the organization (or in this case the team). Voice quality was thus measured as a dichotomous variable with low (option A) and high (option C) quality.

## Results study 1

**Fig 1:**
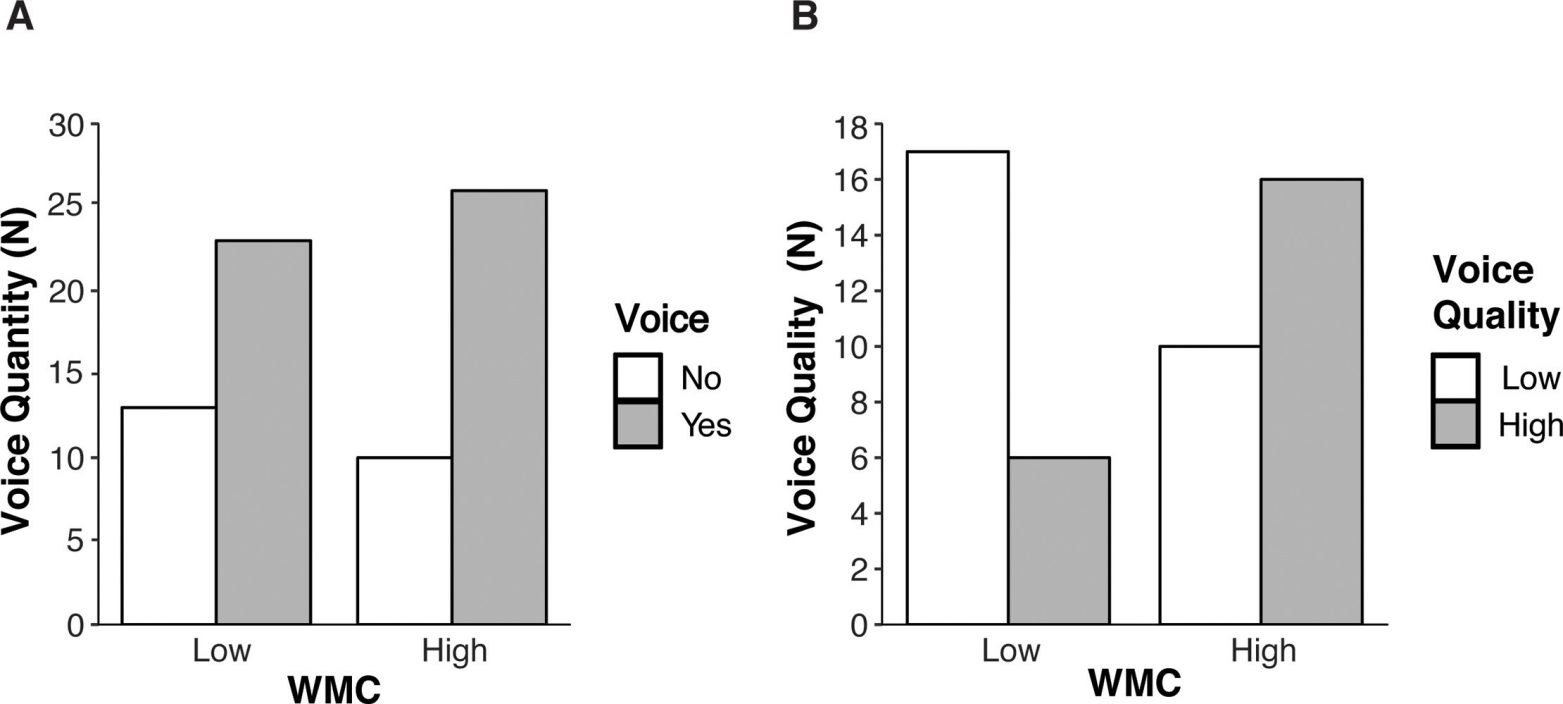
Study 1 –Voice quantity and quality for (low/high) WMC groups. A. Voice Quantity (N participants who voiced / did not voice) by WMC groups (High/Low). B. Voice Quality (N participants who voiced high / low voice quality) by WMC groups (High/Low).

Our data thus consisted of two groups: participants who voiced, and participants who did not voice. Because we hypothesized that WMC is unrelated to voice quantity *(H1)*, we expected no WMC differences between participants who voiced and who did not voice. Indeed, we found no WMC difference between these groups, *F*(71) = .83, *p* = .367, *η^2^* = .012. Within the group who voiced, there were participants who voiced low and high quality. Because we hypothesized a positive relationship between WMC and voice quality *(H2)*, we expected that WMC would be highest in the group who voiced high quality. As predicted, WMC was higher in the group who voiced high quality (*N* = 22, *M* = 5.65, *se* = .16, *CI*_*95*_ *=* (5.32, 5.98)) than in the group who voiced low quality (*N* = 27, *M* = 5.14, *se* = .16, *CI*_*95*_ *=* (4.81, 5.47)), *F*(1,47) = 4.92, *p* = .031, *η^2^* = .095. To visualize these effects, we split the sample into a Low- and High WMC group (using the median: 5.33). The Low WMC group did not voice more than the High WMC group (Fig 1A, *χ^2^* = .58, *p* = .448), whereas the High WMC group voiced more high than low quality solutions, and Low WMC group voiced more low than high-quality solutions (Fig 1B, *χ^2^* = 6.20, *p =* .01). This supports the idea that WMC is related to voice quality *(H2)*, but not to voice quantity *(H1)*.

## Methods study 2

### Ethics, design and sample

Study 2 was conducted in 2016, according to the principles expressed in the Declaration of Helsinki. Informed consent (written) was obtained from all participants. All data was examined anonymously. Participants were aware that they could terminate their participation at any time, and retract their responses within 7 days after participation. Participants were first paid, and then debriefed. The data were collected over a period of 4 weeks. The same experimenter was responsible for data collection during the full data collection process. Below, we provide a short summary of our methods, a more detailed methods section is available at dx.doi.org/10.17504/protocols.io.tubensn.

In order to show that our findings are not limited to between-person differences, but that they are similar within-persons when elaborate cognitive processing is either possible or limited, we designed another experiment using a within-participant design. One of the ways to study causal effects of WMC, is to put load on working memory. Taxing working memory by another activity (such as, for example, counting backwards), reduces its capacity to be used by another activity (for example, voice). We thus designed two within- person conditions in this experiment, one with, and one without distraction of working memory. All participants got the opportunity to voice in both conditions. Further, to assess voice quality, after the experiment, all voice was rated by three raters in terms of whether or not voice provided novel solutions or useful implementations superior to the status quo.

Participants were 77 (we aimed for 80, but lost 2 participants due to incomplete data, and 1 because average idea generation was more than 2.5 *SD* below the mean) primarily business (55%) and psychology (36%) students (67% female, Age *M* = 21.20, *SD* = 3.08) who participated in exchange for money. They were welcomed by the experiment leader and seated behind a computer. The experimenter explained that payment depended on task performance and that performance would be monitored and evaluated at distinct moments in the experiment.

### Procedure, manipulations, tasks, and measures

Participants could engage in two types of payment dependent behaviors. Their primary job was the working memory load task (counting backwards), always resulting in a €2.50 salary if performed correctly. Secondary to this task, there were (extra-role) opportunities to voice, which could result in €1,- bonus (for high quality) or €1,- loss (for low quality) for every voiced suggestion, problem, or opinion depending on the leaders evaluation of voice quality. The experiment started with an exercise of the working memory-load task (counting backwards from 100, in steps of 2) followed by performance feedback. We explained that this task would be repeated several times during the experiment, and that this would always be their primary task with a standard reward of €2.50.

#### Idea generation phase

Our main aim was to find out whether WMC is only important in the idea generation phase as found in previous research [65], or also important in the ‘selection for communication’ phase of voice. To make sure participants would have a pool of messages to choose from, they first anonymously generated 10 problems, opinions, or suggestions for improvement of their faculty [82] in a period of 10 minutes (for the remainder of the text, we call these: ideas). We told them explicitly that these ideas would not be evaluated or used for analysis, but that this was just an anonymous practice round. We needed 10 ideas to be able to fill both conditions (No WMC Load–High WMC Load) with 5 of the participants’ own ideas to potentially voice. During generation, the experimenter digitally classified the ideas (low/high quality). The computer used these classifications for random-weighted message distribution across the WMC Load conditions so both conditions contained an equal amount of high and low-quality messages.

#### Practice phase with simultaneous load manipulation

The generation phase was followed by two combined load and selection practice tasks. We designed two tasks (one simple, one more complex) in which participants needed to select items from a pool of 10 words (the 3 largest animals) or 10 sentences (5 true statements about animals) while they performed a working memory load task (counting backwards). The purpose of these tasks was to familiarize participants with the combination of counting while selecting messages for voice, limiting possibilities for technical confusion during the real opportunity for voice.

#### Communication (voice) phase and simultaneous load manipulation

Following the practice phase, participants got the instructions that they would get to see a random selection of their own ideas, as well as a random selection of other peoples’ ideas. We added 5 ideas from a database of relevant ideas to each Load condition because voice takes place in a social context. In the social environment, the evaluation of the quality of one’s own ideas also depends on the evaluation of the ideas of others. These other-ideas were kept constant between participants: each Load condition contained the full quality (low–high) range of ideas as coded by two independent judges. During the ‘window for voice’, participants thus saw 10 messages from which they could select those ideas they found ‘worthy’ to voice: 5 of their own, and 5 generated by others.

We told them that they would get the opportunity to voice any idea to the experiment-leader, who would evaluate it and pay them accordingly. Voice (as opposed to the generation phase) was thus no longer anonymous and moreover, financially risky: voicing a good idea would result in a €1,- gain, voicing a bad idea in a €1,- loss. Participants were not pushed to voice any ideas, because their primary and most profitable task was to count backwards, either from 80 in steps of 2 (High Load) or from 100 in steps of 0, thus repeating the same number vocally (No Load). Participants performed two trials with 10 messages each and a random order of High vs. No Load. Thus, participants had the opportunity to voice their previously generated ideas, *while* performing a WMC-load task. Voicing ideas was done digitally, by selecting and sending the message to the experimenter, who would evaluate the idea and calculate payment accordingly.

#### Dependent variables voice quality and quantity

*Voice quantity* was measured by counting the number of voiced ideas in each Load condition. Specifically, we counted how many suggestions, problems, or opinions participants sent to the experimenter (quantity) to be evaluated. Across conditions, participants voiced 2.87 ideas on average (*range* = 0–10, *SD* = 2.29). Further, whereas Study 1 measured voice quality as an objective operationalization of value and insight, we now used expert ratings of voice quality. After the experiment, all ideas from the generation phase were evaluated by three independent judges, who rated whether the ideas provided novel solutions or useful implementations superior to the status quo, on a scale from 0 (not at all) to 6 (highly) (adopted from [83]). Inter-rater reliability was reasonable for the generated quality (*ICC =* .*659*). Voice quality was the average quality of those ideas that were voiced by the participants, which was higher (*M*_voiced_ = 3.06, se = .036, _*within*_*CI*_*95*_ *=* {2.99,3.13}) than the quality of the generated ideas (*M*_generated_ = 2.86, se = .036, _*within*_*CI*_*95*_ *=* {2.78,2.93}), *F* (1,64) = 7.76, *p* = .007, *η^2^ =* .108) Within-participant confidence intervals were calculated using the method developed by Loftus and Mason [84], as revised by Cousineau [85]. The same method will be used to report all within subjects 95% confidence intervals throughout the rest of the paper.

## Results study 2

We ran all main analyses using 2-level (No-Load vs. High-Load) repeated measures ANCOVA’s, controlling for the order of the load tasks (No-Load or High-Load first). Furthermore, as both parts of voice are intertwined (voice quality cannot be evaluated without voice quantity), we controlled for voice quantity when testing WMC’s effect on voice quality, and for voice quality when testing WMC’s effect on voice quantity. Voice quality was entered as a continuous standardized covariate when testing for load effects on voice quantity (*H1*). Please note that since some participants never voiced, we could not compute their voice quality, which is modeled as a covariate. This influences the degrees of freedom. Similarly, we controlled for voice quantity, entered as a continuous covariate, when testing for load effects on voice quality (*H2*). We expected that load would not influence voice quantity, and thus that average voice quantity would be the same across the Load conditions. Indeed, we found little difference in voice quantity between the Load conditions. In the No Load condition, participants voiced slightly more (*M*_*No*_ = 1.54, *se* = .082, _*within*_*CI*_*95*_ *=* {1.38, 1.71}) than in the High Load condition (*M*_*High*_ = 1.34, *se* = .082, _*within*_*CI*_*95*_ *=* {1.18, 1.51}), but this effect was not significant, *F* (1,61) = .270, *p* = .61, *η^2^ =* .004.

To increase power, we repeated the analysis using the average generated quality as a covariate. Power increases because also people who did not voice (but did generate ideas) can be included in the analysis. Further, the fact that idea generation happens before voice, is of importance because it might be that people with good ideas just have better ideas to choose from, and therefore voice more. This idea is supported by the finding that idea generation quality was positively related to overall voice quantity, *r* (78) = .38, *p* = .001. Controlling for idea generation quality did not change the effect of WMC Load on voice quantity: (*M*_*No*_ = 1.53, *se* = .069, _*within*_*CI*_*95*_ *=* {1.39, 1.67}), (*M*_*High*_ = 1.36, *se* = .069, _*within*_*CI*_*95*_ *=* {1.22, 1.50}), *F* (1,72) = .61, *p* = .438, *η^2^ =* .008. Similar to Study 1, this suggests that the involvement of WMC in deciding whether to voice and how often to voice, is limited.

In contrast, we did expect that load would influence voice quality, and thus anticipated a difference in voice quality between Load conditions. We expected higher quality of voiced ideas in the No Load condition compared to the High Load condition, because load on working memory should impair elaborate processing and should thus impair effective selection of high-quality ideas. As expected, we found the predicted load effect on voice quality. Again, we controlled for voice quantity because quantity and quality are theoretically intertwined (no quality can be assessed without quantity). Participants voiced lower quality when under High Load (*M*_*High*_ = 3.03, *se* = .054, _*within*_*CI*_*95*_ *=* {2.92, 3.14}), compared to when under No Load (*M*_*No*_ = 3.19, *se* = .054, _*within*_*CI*_*95*_ *=* {3.08, 3.30}), *F* (1,41) = 8.87, *p* = .005, *η^2^ =* .178. Similar to Study 1, this suggests that WMC is important for deciding *what* to voice. However, the fact that the main effect was only significant when controlling for quantity, suggested that there might be an interaction effect.

**Fig 2:**
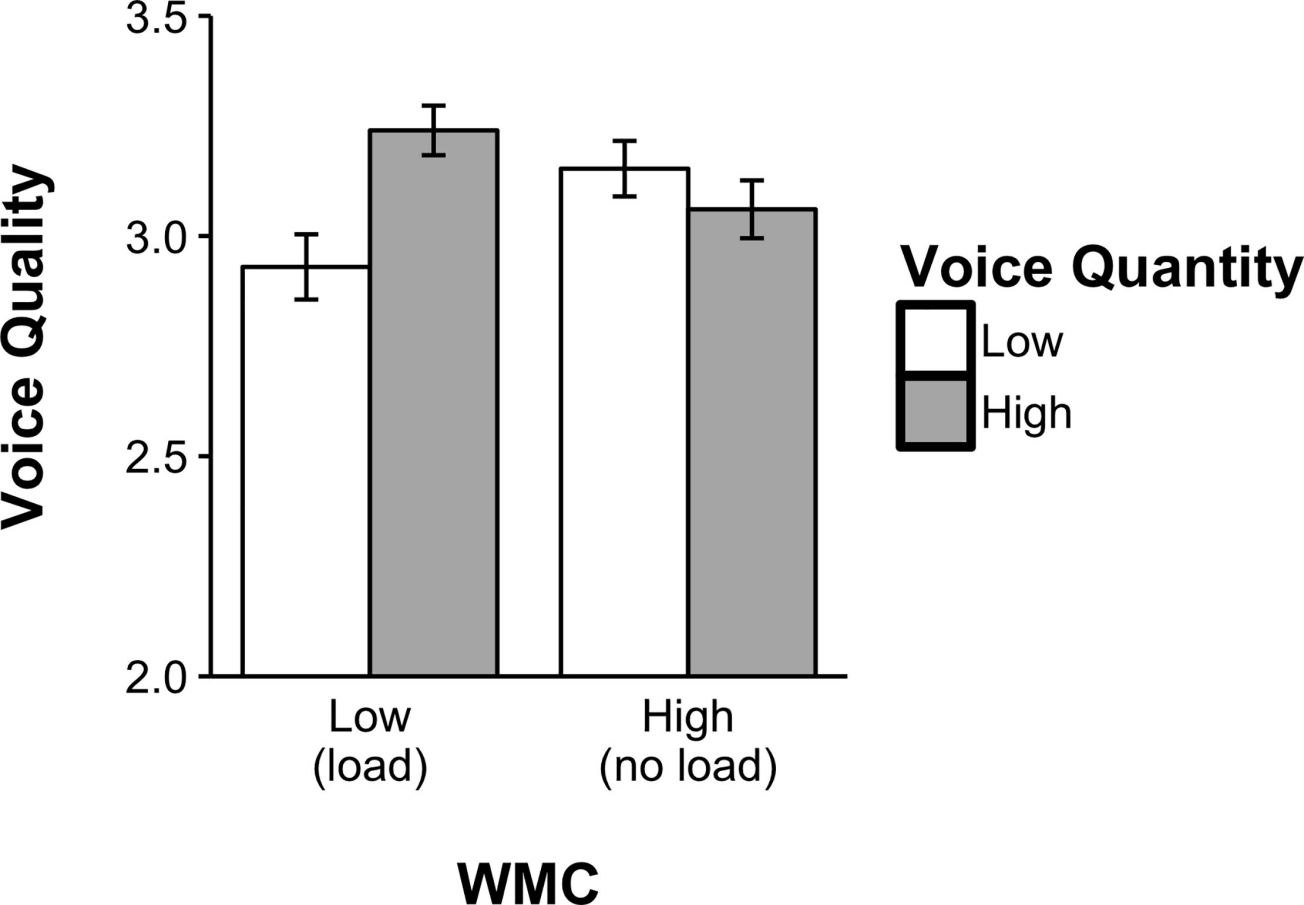
Interaction WMC * voice quantity on voice quality study 2.

Looking at the correlations between voice quantity and voice quality in the different Load Conditions, we found a strong relationship between overall voice quantity and voice quality in the High Load condition (*r* (52) = .40, *p* = .004), but not in the No Load condition (*r* (60) = .01, *p* = .968). Also, we found a significant interaction between WMC Load and overall voice quantity on voice quality, *F* (1,41) = 6.88, *p* = .012, *η^2^ =* .144. To shed light on the direction of this effect, we split (based on the median: 3) our sample into participants who voiced little (3 times or less, *N* = 21) and participants who voiced a lot (4 times or more, *N =* 24). We observed that there was no effect of load (*MΔ*_*NO-High*_ = .09) in the group who voiced a lot, *F* (1,23) = .52, *p* = .480, *η^2^ =* .022. Frequent voicers showed equal voice quality under High Load (*M*_*High*_ = 3.16, *se* = .075, _*within*_*CI*_*95*_ *=* {3.00, 3.31}) and No Load (*M*_*No*_ = 3.06, *se* = .075, _*within*_*CI*_*95*_ *=* {2.91, 3.21}). In contrast, participants who voiced little showed impaired voice quality due to load (*MΔ*_*NO-High*_ = .44), *F* (1,20) = 6.05, *p* = .023, *η^2^ =* .232. For infrequent voicers, voice quality was significantly lower in the High Load condition (*M*_*High*_ = 2.89, *se* = .080, _*within*_*CI*_*95*_ *=* {2.73, 3.05}) than in the No Load condition (*M*_*No*_ = 3.33, *se* = .080, _*within*_*CI*_*95*_ *=* {3.17, 3.49}). It thus appears that WMC is particularly important for voice quality of those who voice little (Fig 2). Please note that the degrees of freedom differ in these analyses because there were people who did not voice at all in either of the two conditions.

The results of this within-participant experiment support the idea that WMC is uninvolved in voice quantity (*H1*), but is involved in voice quality (*H2*). The findings suggest that WMC is not only related to voice quality because it fosters idea generation (as found in previous research), but also because WMC helps individuals to select the best ideas, solutions, problems, and opinions to voice. The interaction effect between voice quantity and working memory load suggests that there might be differences in the involvement of WMC between people who voice often and people who voice little. Presumably, people who voice often, do so more automatically and frequently in general. They might be more familiarized with cognitively evaluating and selecting ideas due to practice and therefore need less WMC to do so. In contrast, people who do not voice often, are not experienced at the evaluation and selection process, and therefore need more space in working memory to do so. In the third study, we set out to replicate our findings in a field setting and to test the idea that the role of WMC differs for those who voice often and those who voice rarely.

## Study 3

Our next step in investigating (WMC) differences between voice quantity and quality, was to test our hypotheses in a field setting. The first challenge was to create a survey measure that would clearly distinguish between voice quantity and quality. Voice measures such as the often used one developed by Van Dyne and Le Pine [1] seem to tap more into the frequency of voice, than its quality. We tested this assumption in a pilot study among 53 employees and their supervisors, by comparing the voice measure by Van Dyne and LePine [1] to several newly developed voice quantity and quality items. We found that the traditional voice scale was only significantly related to voice quantity but not to voice quality (please see the supplementary materials for a full report on this pilot study).

In the following phase, we developed the measures further in order to be able to clearly differentiate between voice quality and quantity in the field. We propose that this distinction is relevant because these two components are (in part) driven by different antecedents, and in particular show differential relationships with WMC. We also explored the relationships of voice quality and quantity with other proactivity constructs, such as proactive personality [86], personal initiative [87] and taking charge [88], and more generally performance. The results of this multi-source pilot field study are reported in the supplementary materials (Table F in S1 Appendix) which presents the nomological network of voice quality and quantity. In summary, the results indicate that voice quality is more strongly related to performance (rated by managers), personal initiative (rated by colleagues), and taking charge (rated by managers) than voice quantity. Further, our Studies 1 and 2 already confirmed the relevance of this distinction for more objective measures of voice quantity and quality specifically concerning WMC.

The first goal of our field study (Study 3) was therefore to replicate those findings in organizations with our further developed voice quantity and quality scales using evaluations of supervisors and coworkers. Our second goal was to investigate whether the reliance on WMC and thus the involvement of cognitive processing in voice quality, might differ between people. Our previous study (Study 2) showed that infrequent voicers were more dependent on WMC to reach high-quality voice than those who voiced a lot. There could thus be individual differences in the way people cognitively approach the act of speaking up. Some people may think elaborately before they voice, organizing thoughts beforehand. Others may be more prone to speak while they think, improvising on the way. Presumably, people get better at selecting and communicating good ideas through practice, and practice (voice quantity) should thus decrease the involvement of WMC in voice quality.

To test this idea, we draw on examples from literature on creativity in musical improvisation WMC and other measures of controlled cognitive elaboration seem to be important for creative performance *only* when musicians are not skilled at improvisation (classical musicians). Those who are highly skilled at improvisation (jazz musicians) are able to perform creatively without it. In classical music undergraduates, high WMC predicted creative performance over time [48]. In contrast, in jazz, undergraduates WMC was related to idea generation (something they were presumably not trained at), but not related to creativity of the improvisational performance [89]. Brain research shows that the right dorsolateral prefrontal cortex, which has been linked to working memory processes in earlier research [90–91], is activated during improvisations of professional classical concert pianists [92]. In contrast, jazz-experts show decreased activity in brain-areas that are associated with consciously monitoring goal-directed behaviors [93]. These findings support the idea that practice reduces the role of WMC in creative performance, and that WMC might serve to overcome a lack of experience in improvising by increasing creative quality of the improvisation through cognitive elaboration.

Extensive training in a creative domain thus seems to be associated with different patterns of brain activation [94]. In sum, creating novel musical patterns may either be achieved through persistent cognitive elaboration (supported by WMC), or by more relaxed flexible and intuitive cognitive processing (supported by improvisational practice). We propose that these findings generalize also to other innovative behaviors, and voicing works in a similar vein. Thus, people who voice often may be less reliant on their cognitive resources to reach high-quality voice as they may have learned how to voice well and usefully in a given context. Those who do not voice often or who have little experience in a given context need to organize all information in their mind and focus attention on the task at hand to be able to speak up with a good comment, and they need WMC to do that. This implies that in practice, there may be two routes to voice quality: A cognitive elaboration route facilitated by WMC, and an intuitive improvisation route that is paved by voice experience (and independent of WMC). We thus hypothesize:

*H3—Voice quantity moderates the positive relationship between WMC and voice quality such that the relationship between WMC and voice quality is strongest if employees voice rarely*.

## Methods study 3

### Ethics, design and sample

Study 3 was conducted in January and February of 2014, according to the principles expressed in the Declaration of Helsinki. Informed consent (written) was obtained from all participants. All data was examined anonymously. Participants were aware that they could terminate their participation at any time, and retract their responses within 7 days after participation. Participants were not paid but participated voluntarily. We offered them a visual presentation of the full research project, and the opportunity to request their personal scores compared to the full sample in a personal visual representation after the study was finished. The data were collected over a period of 8 weeks. Participants were 158 employees ranging in age between 16 and 61 (*M* = 33.57, *SD =* 12.33) and their 79 supervisors who were 41.92 old on average (*range* = 20–65, *SD* = 11.81). The participants worked in a variety of organizations such as SMES (39.7%), consultancy (17.8%), retail (15.8%), governmental institutions (4.8%), non-profit (6.8%), education (6.8), healthcare (8.2%). Gender was equally distributed in the employee sample (50/50), the majority of supervisors were male (62/38). Average employee tenure was 6.7 years (*SD =* 8.62 ), for supervisors, it was a little longer (*M =* 11.2, *SD =* 10.5 ).

### Procedure, tasks, measures

We collected visual WMC and voice data from 158 employees in 79 teams of unique matched triads. Each employee was tested for visual WMC, after which they completed a questionnaire to rate one of their colleagues’ voice quantity and quality (and the colleague did the same about the focal employee). The team-supervisor (*N* = 79) rated both employees on voice quantity and quality. All tests and questionnaires were administered with a researcher present. Researchers made an appointment with each team, personally explained and distributed all measures, debriefed participants, and left after all data were collected.

#### Independent variable WMC

Participants first took a visual WMC test (programmed in Presentation version 17.3) which was a combination of change localization and storage tasks which has been used in previous research on visual WMC [85] . There were three different basic tasks: change localization, serial spatial memory, and simultaneous spatial memory. All basic tasks were executed with pure storage attributes as well as distractor interference resulting in 8 different types of trials. In all trial-types, participants had to store objects (circles and rectangles) in their working memory (remember them). During this target phase, objects were displayed for 250 ms in a 5x5 grid. The location of all objects should be remembered during a delay period of 500 ms after which they disappeared. Objects were green (targets) and yellow (distractors) rectangles (120x40 pixels, 0.89 visual degree) and circles (diameter 120 pixels, 2.68 visual degree). After a 500 ms delay (objects disappeared), participants either had to recall the location of all circles or localize one rectangle that changed. The test started easy (1 object), adding objects gradually over trials, and only ended when the individual capacity limit within all trial-types was reached. The test was reliable (*α* = .73). WMC was calculated using Cowans’ K, a capacity estimate for tasks that require participants to remember multiple items in one visual field, as opposed to the serial task we used in study 1 [95,96].

#### Dependent variables voice quality and quantity

Colleagues and supervisors rated focal employee voice quantity (15 items) and quality (15 items) with a questionnaire (Tables C and D in S1 Appendix). To control whether raters understood the behavior we intended to measure was *a)* discretionary, proactive, and constructive and *b)* distinct in the sense that quantity does not imply quality and vice versa, we first explained this followed by control questions. Voice quality was meant to reflect novelty and usefulness of voiced information (suggestions, problems, and opinions). Voice quantity was reflected in the frequency of voiced information (suggestions, problems, and opinions). One voice quantity item was deleted due to poor factor loadings. Cronbach’s’ alphas were high for colleague (α _quality_ = .93, α _quantity_ = .89) and supervisor ratings (α _quality_ = .96, α _quantity_ = .91). Descriptives and correlations can be found in Table E in S1 Appendix.

#### Measurement model

Our first aim was to replicate our pilot-study findings (Tables A and B in S1 Appendix) that voice quality and quantity are separate constructs. If voice quality and quantity are indeed distinct, a 4-factor (2 sources x 2 types) measurement model should fit the data better than a 2-factor (2 sources) model which represents voice as one construct. We tested this with a CFA in Mplus. As expected, the 4-factor model with a separation of quantity and quality provided a good fit (*χ^2^* = 167.07, *df* = 101, *N* = 144, *p* < .001, CFI = .97, TLI .96, RMSEA = .07, 90% CI {.05, .08 }, AIC = 4055, BIC = 4206). We compared this model to a 2 factor-model, with quantity and quality as one factor, and the source (colleague vs. supervisor) as separate factors, which showed a significantly poorer fit (*Δχ^2^* = 397.53, *df = 2*, *p* < .001), (*χ^2^* = 564.60, *df* = 103, *N* = 144, *p* < .001, CFI = .78, TLI = .74, RMSEA = .18, 90% CI {.16, .19 }, AIC = 4448, BIC = 4594). This again suggests that voice quality and quantity are distinct constructs, not only when rated by supervisors, but also by colleagues. More details and in-depth analyses can be found in the supplementary materials (S1 Appendix), factor loadings (range .74 –.95) and scale details can be found in Tables C and D in S1 Appendix.

## Results study 3

In the previous studies, we showed that WMC relates to the quality of voice *(H2)*, but not the quantity of voice *(H1)*. Although our hypotheses are at the individual level, our study has a two-level data structure, with employees nested in 3-person teams with one supervisor. Employees rated each other, whereas supervisors rated both employees. To take between-group and within-group variance into account when using combined ratings of colleagues and supervisors, we applied multilevel structural equation modelling (MPlus) to control for team-level variance. Since our measurement model indicated that colleague and supervisor ratings are distinct factors and the literature implies the same, we first tested our hypotheses in separate multiple regressions.

The regression analysis of our first model (colleague ratings) showed no significant effect of WMC on the quantity of voice (*β*_*controlled for quality*_ = .02, *p* = .832, *se* = .07, *β*_*simple effect*_ = .10, *p* = .225, *se* = .08) whereas the effect of WMC on voice quality was positive (*β*_*controlled for quantity*_ = .14, *p* = .04, *se* = .07, *β*_*simple effect*_ = .18, *p* = .016, *se* = .08). Employees with high WMC thus voiced better suggestions, opinions, and problems to their colleagues than employees with low WMC *(H2)*. However, they did not voice more often *(H1)*. As expected, we found the same pattern of results for supervisor ratings. Employee WMC did not significantly predict voice quantity (*β*_*controlled for quality*_ = -.03, *p* = .622, *se* = .05, *β*_*simple effect*_ = .12, *p* = .095, *se* = .07), but did positively predict voice quality towards supervisors (*β*_*controlled for quantity*_ = .13, *p* = .033, *se* = .06, *β*_*simple effect*_ = .21, *p* = .011, *se* = .08). Results for the combined sources show the same pattern: WMC is unrelated to voice quantity (*β*_*controlled for quality*_ = -.04, *p* = .418, *se* = .05) and positively related to voice quality (*β*_*controlled for quantity*_ = .15, *p* = .005, *se* = .05). The capacity of working memory thus seems to be important for generation, selection, and communication of high-quality voice, regardless of the voice target.

**Fig 3:**
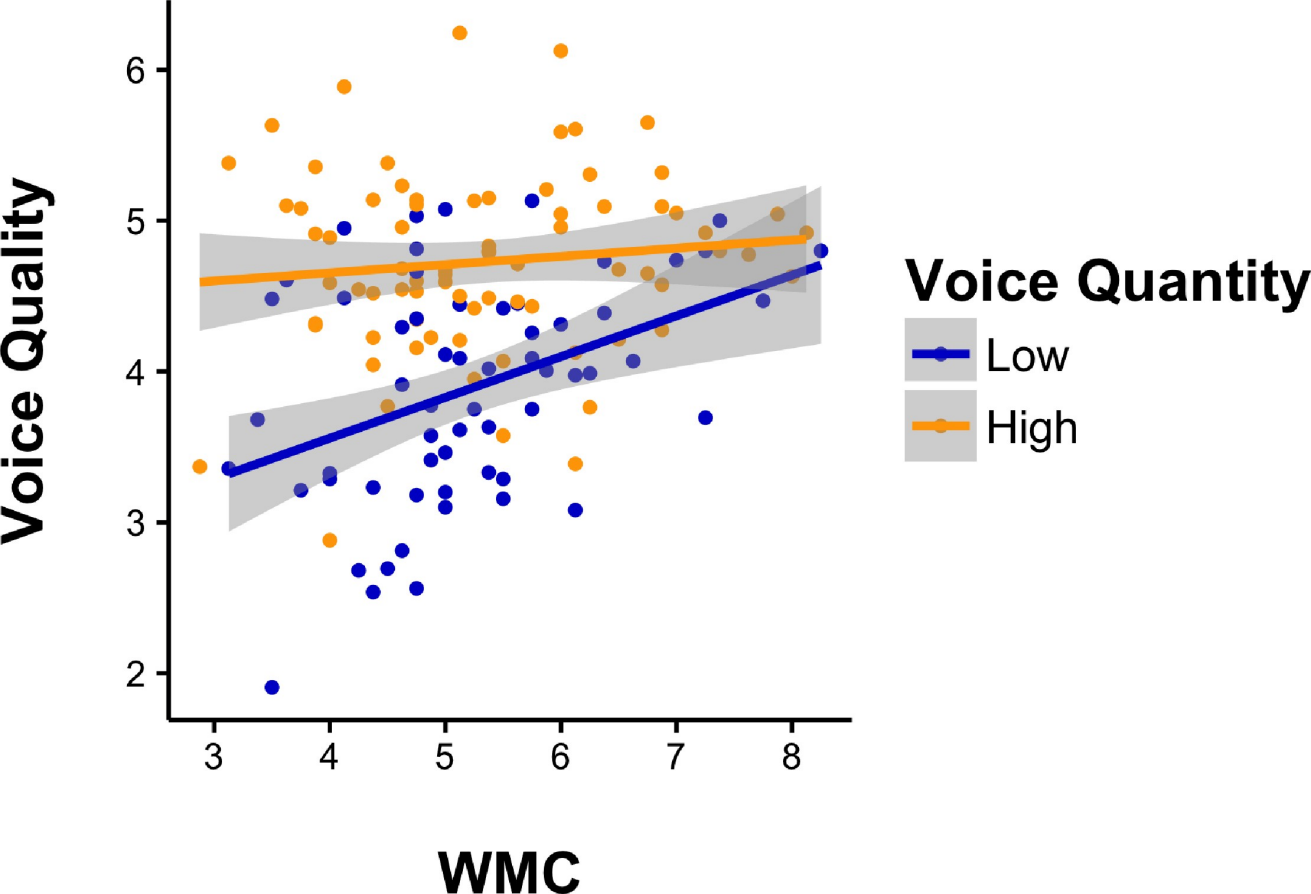
Interaction WMC * voice quantity on voice quality study 3.

We hypothesized that due to practice, people who typically voice often rely less on WMC than people who tend to voice rarely *(H3)*. Consequently, we expected negative interactions between voice quantity and WMC when predicting voice quality: WMC should have the strongest positive effect on voice quality when voice quantity is low. We first tested this assumption concerning all ratings of voice (supervisor and colleague ratings combined) in MPlus using a robust maximum likelihood estimator. We started with the total model (both sources), and continued with robustness tests with any possible combination of sources in the model. If practice indeed increases automaticity of voice and in turn decreases the influence of cognitive resources (WMC), this pattern should exist in every model, regardless of the source rating quantity and quality. As expected, we found a negative interaction effect (*β* = -.17, *p* = .003, *se* = .06 ). Simple slope analysis (based on a split through the mean) showed that the relationship between WMC and voice quality was positive in the group who voices little (Low_*Quantity*_: *β* = .40, *t =* 3.33, *p* = .001) and non-significant in the group who voices often (High_*Quantity*_: *β* = .15, *t =* 1.30, *p* = .199). This interaction (Fig 3) is in line with our findings in Study 2 (Fig 2), that WMC load affected voice quality only for participants who voiced little. Next, we proceeded with the same analyses for every possible combination of sources. We consistently found the same pattern of results in all models, which can be seen in Table 1.

**Table 1:** All interaction models with dependent variable voice quality.

DV Voice Quality	Independents	*β*	*se*	*p*
**Total**	Voice Quantity Total	.65**	.06	.000
	WMC	.17**	.05	.001
	Quantity*WMC	-.17**	.06	.003
	Voice Quantity to Supervisors	.66**	.05	.000
**Supervisor Ratings**	WMC	.14**	.05	.008
	Quantity*WMC	-.14**	.05	.007
	Voice Quantity to Colleagues	.44**	.09	.000
**Colleague Ratings**	WMC	.15*	.07	.029
	Quantity*WMC	-.23**	.07	.001
	Voice Quantity to Supervisors	.33**	.09	.000
**Colleagues Ratings**	WMC	.17*	.07	.017
	Quantity*WMC	-.22*	.10	.040
	Voice Quantity Colleagues	.30**	.07	.000
**Supervisor Ratings**	WMC	.18*	.08	.018
	Quantity*WMC	-.12†	.06	.055

Note.

† *p* < .10

* *p* < .05

** *p* < .01

*** *p* < .001.

**Fig 4:**
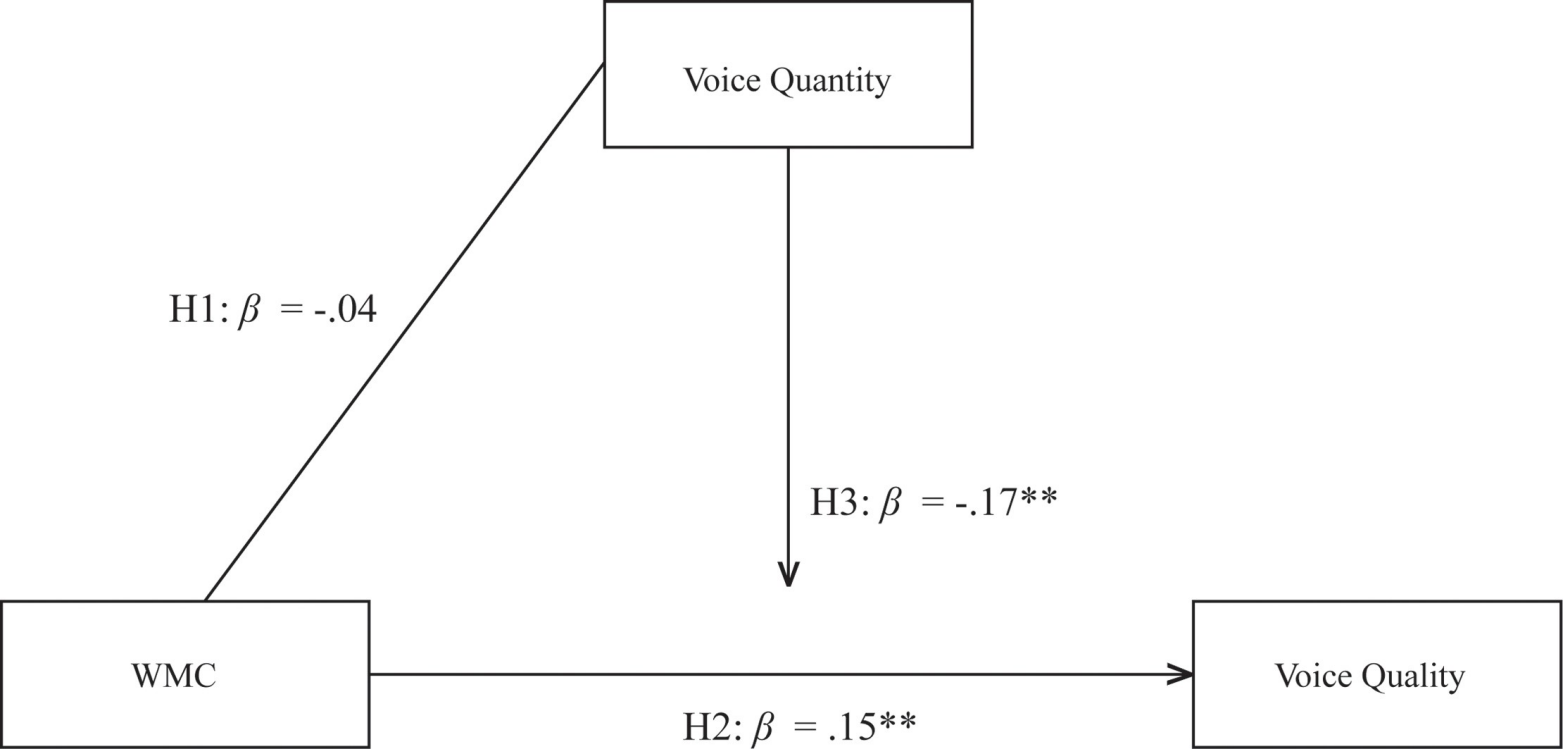
Model summary of all hypothesized effects study 3.

To summarize all results of Study 3, in Fig 4, we provide a visual overview of all hypothesized direct and interaction effects. This model shows that WMC relates to voice quality *(H2)*, but not voice quantity *(H1)*, and that quantity of voice (which is positively related to quality), moderates the relationship between WMC and voice quality *(H3)*.

## General discussion

This paper aimed to incorporate knowledge from experimental psychology and cognitive science to further our understanding of the cognitive processes underlying voice behavior in organizations. We proposed and found that voice quality and quantity differ with respect to their relationship with elaborate cognitive processing in terms of WMC. As hypothesized, the results show that voice behavior in and of itself (i.e., voice quantity or frequency) does not require either the ability (Working Memory Capacity) or the opportunity (no load on Working Memory) to engage in elaborative thinking. Generating, selecting, and communicating high-quality messages to voice (voice quality), however, does require cognitive elaboration, especially for people who rarely voice.

First, our results showed that voice quantity required less cognitive elaboration than voice quality. Voice is socially risky behavior, and previous research has shown that assessment of risk is often not rational, but rather based on affect and heuristics [52,97,98]. When people decide to voice, they might just do what felt good in the past, relying on social schema’s and affect and thereby leaving cognitive elaboration unnecessary to decide whether to voice or not. Even though voice is a proactive behavior, and proactive behavior is assumed to be discretionary and agentic, the existence of constructs like proactive personality [86] and personal initiative [99] indicate that there is a trait-like, automatized component to proactivity that is relatively stable across situations. If people are consistently proactive (or un-proactive), they might not always carefully deliberate whether they want to be proactive or not. Our findings indicate that when it comes to proactive *voice* behavior, this could be the case. Whether (study 1) and how often (study 3) people voice opinions, problems, or suggestions to colleagues or to supervisors was not related to individual differences in WMC, and unaffected by WMC-load (Study 2).

Our findings add to previous research showing that silence is often the result of fast, implicit, and affective reactions, not of careful cognitive elaboration [53,54]. Silence, however is not the exact opposite of voice [100]: where silence means that the employee knowingly withholds information, not voicing could mean that there simply is no information to share. People high in WMC may pick up more information and therefore theoretically may have more to share, which could result in more voice. Our first lab-study however, in which participants had to detect and voice information inconsistencies, showed that WMC was equal between the group who voiced and the group who did not voice. So even if high WMC increases the capacity to detect and solve problems and inconsistencies, this does not mean that this capacity is used, at least not in a short time frame such as our experiment. Our results thus indicate that not only the decision to remain silent, but also the decision to voice might be more automatic than elaborate and cognitively complex.

Based on the relationship between WMC and creativity [27,64], we hypothesized that WMC plays a role in reaching high-quality voice content through facilitating persistent, elaborate thought. The more information one can actively maintain in awareness (storage capacity), and the better one’s capacity to control attentional resources (distractor interference), the more elaborate and complex thinking is possible. This should increase chances to come up with new and useful ideas (creativity), but should also help to evaluate, select and communicate the best information (voice quality). Indeed, we found positive relationships between the cognitive resource WMC and objective (Study 1) as well as multi-source perceptual measures (Study 3) of voice quality. Furthermore, hindering elaborate systematic thinking (through WMC-load, Study 2), impaired the selection of high-quality ideas for voice. People presumably need space in their working memory for voice, but mostly when they try to evaluate and select high-quality messages to convey.

Third, our results also point at individual differences in using elaborate cognitive processing for voice behavior. More specifically, the involvement of cognitive resources in voice quality seems to differ between people who voice often and people who voice little. The data in study 2 and 3 indicates that the involvement of WMC in voice quality is strong for people who voice little, but nonexistent for people who voice often. There are several theoretical explanations for these findings which we discuss below. We start with our main explanation, namely that those who voice rarely rely on cognitive elaboration (and thus WMC), whereas those who voice frequently have learned to improvise high voice quality over time.

### Cognitive elaboration versus improvisation

We argued that voicing often would result in experience with evaluation and selection of messages and with typical reactions from others in a given context. People who voice more than others should thus decrease their reliance on cognitive resources for high-quality voice over time, allowing to become faster and cognitively more effortless at voicing high quality. People who voice little, however, need time and cognitive elaboration to reach high voice quality. These results mirror the findings from creativity research that high WMC musicians only become more creative when they spend longer periods of time on an improvisation [65]. WMC is thus beneficial to those who spend time on elaboration. Frequent voicers might be better able to anticipate reactions of others due to previous experience, and may therefore act more intuitively during conversations, making the advantages of WMC less important to reach high-quality voice.

The fact that to infrequent voicers, voice is more novel than to frequent voicers, is important because of the storage capacity function of WMC. People who voice often may not need the same degree of WMC to form complex connections between concepts, because these already exist in long term memory and automatized patterns. Because they have experience with voice (and thus thinking about what to say), they can chunk more information, making it easier to keep track of previously mentioned information, maintaining quality over time. People who voice little however, cannot rely on long term memory because to them, message evaluation and selection is novel, and thus their storage capacity determines whether or not they are able to voice high quality.

### Alternative explanations

Another, more trait-based interpretation of our results, indicates that people differ in their use of working memory resources because of personality (as opposed to learning). For example, introverts voice less than extraverts [101], but they are not necessarily less creative [102]. Because WMC enhances creativity, high WMC introvert individuals may voice rarely due to their personality, but voice high quality nonetheless because they have a high-quality pool of ideas to begin with. Extraverts may use a quality through quantity route: if one voices a lot of ideas, chances are that one of those ideas will be of high quality may increase. It could also be that some people are generally more creative than others [103], and just have a better quality idea pool to begin with because they spend more time generating ideas in general. These explanations are supported by the fact that the quality of ideas in the generation phase of study 2 was related to the number of voiced ideas in both WMC load conditions. There might thus be a path from creativity, through voice quantity, to voice quality, or from voice quantity to creativity, to voice quality. However, our data does not allow for testing the order of the variables in this chain, and longitudinal research is needed here to better explain this process.

### Strengths, limitations, and future research

#### Sample and experimental design

An obvious limitation of this study is the use of a student sample and of simulated organizational tasks. Although we tried our best to simulate the organizational environment carefully by using an actual social interaction, leaders, evaluations, and (financial) riskiness of voice, the external validity of our two experiments may have suffered from the highly controlled settings. The findings from the two laboratory studies thus require a replication in samples of employees (which we did in study 3), but also in field experiments and in real-time business environments (e.g., a study with recordings of actual voice behavior during meetings [104].

#### Voice measures

Because we wanted to show that our hypotheses hold across different types of research methods, we also used different types of voice measures, which we feel is a strength. We have measured the quantity and quality of voice differently in all studies with the intention of showing that the distinction between voice quantity and quality is not measurement (and thus method) specific. The convergence of the results across all three studies provides evidence for the validity of our theoretical distinction. Yet, we acknowledge that our measure of voice quality in study 1 is closer to decision making quality than our measures of voice quality in Studies 2 and 3. However, we aimed to explore what mentally happens when people decide to voice or not, and what they decide to voice (and what the quality of that ‘what’ is), and our voice measure in Study 1 covers the core aspects of voice even though it incorporates the originality and usefulness of voice elements differently than the measures in Studies 2 and 3.

One other point of note, is that the correlation between supervisor-ratings of voice quantity and quality was rather high (higher than for colleague ratings). This could indicate that supervisors are less able or inclined to separate the two. It could also imply that if employees choose to voice they may be more selective in what they voice to supervisors compared to what they voice towards colleagues. First, if an employee feels that s/he has something valuable or useful to say, the likelihood of voicing to a supervisor presumably increases. Second, if voice quality towards supervisors is low, negative feedback might influence quantity of voice more severely than in the case of voice towards colleagues. Furthermore, even though the relationship between supervisor rated quantity and quality of voice was relatively strong, WMC was still only related to voice quality, not to voice quantity. So even though voice towards supervisors might be riskier than voice towards colleagues, WMC is not more strongly related to how often employees voice towards supervisors than to how often they voice to colleagues. This is in line with our idea that deciding to voice is not necessarily elaborated because risky decisions rely on automaticity rather than on cognitive elaboration.

#### WMC measures and manipulations

Although our results show the same pattern across three studies, we have to point out that we used different types of WMC measures in each study and that the theoretical implications might be slightly different for each study. In our first experiment, we used a visual WMC measure that may not capture only working memory capacity. Because the task requires participants to *recognize* whether or not an object was present in the previous series, part of the score may reflect long term memory effects on top of WMC. This might explain why the mean scores were slightly higher than in Study 3, in which we used another measure of WMC based on recalling objects’ locations, as opposed to recognizing whether an object was previously presented. This mean difference, however, could also be explained by the fact that Study 3 was an employee sample whereas Study 1 was a student sample (which is supported by the fact that standard deviations were larger in Study 3). Still, it might be the case that in Study 1, better long-term memory played a role in participants with high WMC scores voicing better quality ideas.

Ideally, in study 2, we should have measured individual differences in WMC on top of our Load Manipulation. The complexity of the design forced us to make additional tasks as short as possible and thus limited us in adding individual differences in WMC as a covariate. Furthermore, although the literature supports the idea that our manipulation in Study 2 put load the Capacity of Working Memory, this remains an assumption. Since the use of WMC requires attention control [71–73], distracting attention with another task should (partly) limit the use of WMC [65,70,105]. However, a more conservative conclusion of the results in Study 3 would be that distracting attention from voice limits the quality of those who voice rarely. Nevertheless, we found the same interaction in Study 3, indicating that this effect is likely not only driven by attention and distractor interference, but also by the storage component of WMC.

#### Future research

We tested our core assumptions about the relationship between WMC and voice quality and quantity in three different designs: a between-subjects lab design, a within-subjects experimental design, and a multi-source field study. The proposed (un)involvement of WMC in quantity and quality of voice was found and replicated in all three studies. Our study design only allowed us to test (and confirm) the negative interaction between WMC and voice quantity in Studies 2 and 3. It would be interesting to replicate and extend this using multiple indicators of experience and personality. Investigating differences in WMC involvement during voice for employees whose core task involves innovation and from other (non-innovation trained) departments, between teams who had proactivity training or not [106], between individuals from teams with differing climates for voice [45], or people high and low in personal initiative, would increase in-depth insight into this interaction.

Furthermore, over time, WMC might influence voice quantity through quality. Even if voiced rarely, positive responses to high-quality voice will likely enhance voice quantity in the future. Investigating mediation processes between WMC, voice quality, and voice quantity, and moderating effects of different leadership styles would benefit our understanding of what the ideal climate for voice quality is.

Finally, it is not yet clear whether WMC facilitates voice quality through storage capacity, distractor interference, attention, or all of them. Further research on these separate functions of WMC might be beneficial to better understand how WMC aids voice. Furthermore, there are other cognitive resources that are likely related to WMC, such as inhibitory control [107]. The ability to inhibit or control automatically triggered responses could be important for voice quality in other ways than through focusing attention on voicing. For example, previous research has shown that voice is more effective when employees have the ability to regulate their (negative) emotions [108]. Because voice may sometimes occur as a response to a problem or dissatisfaction, the ability to inhibit responding until one has developed something truly useful to say, may benefit chances that voice will be valued.

#### Implications for practice

The findings of our studies offer important implications for practice. Cognitive neuroscience has shown that visual WMC might be a fundamental basis for complex thinking [24,109]. Visual working memory tests are faster, cheaper, evolutionarily more basic and less culturally biased than the more general mental ability tests that are common in management literature and selection procedures. They do not require domain specific knowledge and thus do not obstruct people with for example, dyslexia or other language (due to being from a different culture) disadvantages. In terms of enhancing diversity in organizations, this might thus be a better way to assess whether people are able to engage in elaborate thinking.

Regarding our dual pathways to voice quality, there are two important issues to consider for managers (if they wish to enhance quality voice). First, some people (infrequent voicers) need cognitive resources to voice high quality. Measuring WMC before hiring people may thus be a way to select those. However, these people also elaborate to reach high-quality voice, and elaboration takes time and effort. Merely hiring high WMC people is thus not enough, there needs to be time and space to elaborate. Second, some people (frequent voicers) need to be able to practice voice in order to learn how to improvise. Since quantity of voice seems to be more automatic (not well elaborated), trying to influence this process with rational arguments might not be very effective. People either voice often because it is in their personality, or because in their team, there is an implicit feeling that voice is safe. If people in a team generally voice rarely, it may be more useful to try to enhance voice by focusing on the implicit cues the team leader is sending, than explicitly telling people they can always share their concerns.

#### Theoretical contributions

The first contribution of this study is the distinction between voice quality and quantity. It opens up new avenues for studying the multidimensionality of voice behavior and emphasizes the difference between a decision to say something and the quality of what is said. This focus on content relates to both the work on creativity, such as the combination of originality and usefulness for truly innovative ideas [110], and to voice research, such as the (complexity) differences between suggestions, opinions, and problems [2], and the differences between promotive versus prohibitive voice [29]. This paper strengthens the claims of those studies that it is important to consider the content of voice if we want to know more about how effective voice is generated, selected and communicated.

Our second contribution is the investigation of the involvement of cognitive resources in voice behavior. Morrison [2] called for more research in the cognitive elaboration versus automaticity area, which we answered by challenging the idea that voice behavior in and of itself is the result of a complex cognitive calculation. We attempted to sharpen the hypotheses about the involvement of elaborate cognitive processing in voice by showing that the arguments of Chiaburu and colleagues [28] about cognitive elaboration hold, but only for the quality of voice and especially when employees don’t voice often. Our results are also in line with the idea that choosing (not) to voice depends on affect and implicit theories about voice [53]. Our findings thus reconcile both views by showing that indeed, voice quality (sometimes) requires elaborate processing, whereas voice quantity relies on automatic processing. The results of this paper emphasize the importance of a cognitive-processes stream in the voice literature. Automaticity in a self-initiated, discretionary proactive behavior such as voice may seem like a paradox, but often, cognitive elaboration might be less important than we think.

Third, we contributed two different experimental designs (within- and between subjects), which makes a wide range of causal research questions about individual differences as well as situational differences about voice and initiative possible. Although we did not use our first lab-setting for causal purposes, the design does offer that possibility [111]. Since experimental research in voice and proactivity literature is still scarce, we hope that this helps other researchers to tackle causal research questions concerning proactive behavior.

## Supporting information

S1 Appendix Supplementary materials dual pathways to voice quality. (DOCX)
