# Supplementary material for: Dual cognitive pathways to voice quality: Frequent voicers improvise, infrequent voicers elaborate
Source: PLoS One. 2019 Feb 27;14(2):e0212608. doi: 10.1371/journal.pone.0212608 (PMC6392316; doi:10.1371/journal.pone.0212608)
Supplement: S1 Appendix — (DOCX) [file pone.0212608.s001.docx]

**Supplementary Materials Dual Pathways to Voice Quality**

**Pilot Studies Voice Quantity and Quality Scale development**

Before we designed study 3, we gathered data to test and support the idea that the original voice measure that is often used in voice research, taps into how frequent people voice, not how creative, novel, useful, or innovative voice is. We further wanted to test whether quantity and quality were separate constructs before conducting a large field study to connect them to WMC. We thus formulated and tested the following assumptions:

*A1 - Voice quantity and quality are distinct but related aspects of voice.*

*A2 - The commonly used measure of voice is more strongly related to how often employees voice (quantity), than to the content of their messages (quality).*

**Design**

We conducted a multi-source pilot study with unique dyads composed of 53 supervisors (*M*_age_ = 39.9, *SD*_age_ = 8.69, 34% male) and 53 subordinates (*M*_age_ = 36.5, *SD*_age_ = 15.12, 30% male) in a variety of companies (largest groups: 45% retail, 20% banking, 20% healthcare). We compared the measure that is most often used in voice research (Van Dyne & LePine, 1998) to our pilot items of voice quantity and quality (see Appendix, Table S1 for all items). Like in Study 3, digital questionnaires were distributed on location, where participants completed them in the presence of a researcher. We measured self-ratings of *voice* (Van Dyne & LePine, 1998, 5 items, *α =* .71) at employee level, and asked supervisors to rate the quantity (3 items, *α* = .73) and quality (4 items, *α* = .89) of their subordinates’ voice.

**Results**

Since we assumed that voice quantity and quality are distinct but related (*A1*) concepts, we performed an exploratory factor analysis (EFA) with oblimin rotation on the supervisor ratings of quantity and quality. The EFA (*KMO* =.79, 75% of variance, *p* <.001) showed two factors: Quality (*KMO* = .79, 54% variance, Eigenvalue = 3.81) and Quantity (*KMO* = .67, 21% variance, Eigenvalue = 1.50). Factor loadings can be found in table S1). As expected, we found a positive correlation between the two concepts (*r* = .37, *p* = .002). This suggests that supervisors identify voice quality and quantity as distinct constructs, yet they tend to rate voice of employees who speak up often as more original and useful than voice of employees who voice rarely.

|  |  |  |
| --- | --- | --- |
| **TABLE A - Pilot items for Voice Quantity and Quality** | | |
|  | **Factor Loadings** | |
| **Items** | **Quality** | **Quantity** |
|  | **54%** | **21%** |
| If my colleague presents a new product or method, it is carefully thought through | **.93** | -.03 |
| When my subordinate shares an idea, he or she has considered how it should be implemented | **.94** | -.17 |
| If my subordinate shares an idea, it is usually original | **.78** | .13 |
| My subordinate shares ideas that are useful in practice | **.8** | .11 |
| My subordinate regularly communicates ideas that are meant to help the organization develop | .43 | **.59** |
| How many improvement oriented ideas has your subordinate communicated in the past month? | .13 | **.83** |
| How often did you subordinate constructively share his or her opinion with you over the past month? | -.18 | **.94** |
|  |  |  |

Secondly (*A2*), we expected that although the classical voice measure presumably incorporates both quantity and quality of voice, the relationship with quantity is stronger. To test this, we related supervisor-rated quantity and quality of voice to employees’ self-ratings of voice (see Table S2 for descriptives and correlations). Results from a linear regression analysis showed that the self-rated voice scale was positively related to supervisor-rated voice quantity (*β* = .32, *t* = 2.16, *p* = .04 *se* = .06 ), but not voice quality (*β* = .08, *t* = .60, *p* = .58, *se* = .10). Thus, the scale developed by Van Dyne and Le Pine (1998) seems to capture how often people voice, but might be less geared towards predicting whether voice adds novel and useful value to the organization.

|  |  |  |  |  |  |  |
| --- | --- | --- | --- | --- | --- | --- |
|  | **TABLE B - Descriptives and zero-order correlations between voice constructs** | | | | | |
|  |  |  |  | 1 | 2 | 3 |
|  |  | *Mean* | *SD* | **Voice** | **Quantity** | **Quality** |
| 1 | **Voice** | 5.14 | .82 | (.71) |  |  |
|  | (self-rated) |  |  |  |  |  |
| 2 | **Voice Quantity** (supervisor-rated) | 2.99 | 2.2 | .36** | (.73) |  |
| 3 | **Voice Quality** (supervisor-rated) | 3.93 | 1.28 | .24† | .52** | (.89) |
|  |  |  |  |  |  |  |

**Note.** *p* < .05, ** *p* < .01, *** *p* < .001.

**Study 3 Measurement Model of voice Quality and Quantity**

To assess construct validity of our measures in Study 3, we performed a CFA (in Mplus) for voice quantity and quality, while controlling for common method source-bias by indicating the source as a higher order latent factor^[[1]](#footnote-1)^. This resulted in a 4 factor model (2 sources, 2 types). If voice quality and quantity are indeed separable constructs (instead of one voice construct), this measurement model should fit the data best. We randomly parcelled the items of quantity and quality to decrease the number of parameters in our model (Bagozzi & Edwards, 1998; Nasser-Abu Alhija & Wisenbaker, 2006). To make sure all parcels were similar in content, we used the following restrictions: each quantity-parcel contained at least one suggestion, one opinion and one problem focused item and in the case of voice quality an equal distribution of novelty and usefulness items. We simply used the item numbers to further distribute items across parcels, to avoid bias in allocating the items. This procedure resulted in 4 parcels per factor.

As expected, the 4-factor model provided a good fit (*χ²* = 167.07, *df* = 101, *N*=144, *p* < .001, CFI = .97, TLI = .96, RMSEA = .07, 90% CI [ .05 , .08 ], AIC = 4055, BIC = 4206). We compared this model to a 2 factor-model, with quantity and quality as one factor, and the source (colleague vs. supervisor) as separate factors, which showed a poor fit to the data ( *χ²* = 564.60, *df* = 103, *N*=144, *p* < .001, CFI = .78, TLI = .74, RMSEA = .18, 90% CI [ .16 , .19 ] , AIC = 4448, BIC = 4594). A Chi-square difference test showed that both four factor models with a separation in voice quantity and quality, showed a significantly better fit to the data than the model based only on the two sources. Both 4-factor models (either with Voice Type, *Δχ² = 368.66, df =2 , p < .001*) or with Source (*Δχ²* = 397.53*, df =2* , *p* < .001) as the higher order latent factor, showed a better fit to the data than a 2-factor model with only the source to separate the measures. This suggests that voice quality and quantity are distinct constructs both for colleague and supervisor ratings. All factor loadings (range .74 – .95) can be found in the table S3a for Quantity, and table S3b for Quality, along with scale items, and parcel allocations.

Because there was no room for a zero order correlation table of study 3 in the main text, we present it here in table S4. Last, in table S5, we present the results of a separate multi-source field study that was conducted to map the nomological network of voice quality and quantity.

| **TABLE C - All Voice Quantity Items and Parcel Loadings of the 4 Factor- Model** | | | | |
| --- | --- | --- | --- | --- |
| **Rater** | **Parcel** | **Loadings** | **Items** | **Category** |
|  |  |  | **On a scale indicating 1. Never 2. Rarely 3. Sometimes 4. Regularly. 5. Frequently** | |
|  |  |  | **How often does your colleague/supervisor proactively and constructively communicate:** |  |
| Colleague | 1 | .85 | Suggestions for improvement | Suggestion |
|  |  |  | His or her opinion, even if it is different from the view of others | Opinion |
|  |  |  | Remarks that highlight problems in the organization | Problem |
|  | 2 | .85 | Solutions for problems in the organization | Suggestion |
|  |  |  | Advice that deviates from the norm | Opinion |
|  |  |  | His or her opinion regarding issues within the organization | Opinion |
|  |  |  | Observations of misconduct | Problem |
|  | 3 | .78 | New ideas | Suggestion |
|  |  |  | Personal advice | Opinion |
|  |  |  | Concerns about processes that are not functioning optimally | Problem |
|  | 4 | .85 | Plans to change procedures or processes | Suggestion |
|  |  |  | Spontaneous suggestions | Suggestion |
|  |  |  | His or her opinion about the status quo within the organization | Opinion |
|  |  |  | Attention towards inefficiencies in the workplace | Problem |
| Supervisor | 1 | .88 | Solutions for problems in the organization | Suggestion |
|  |  |  | His or her opinion about the status quo within the organization | Opinion |
|  |  |  | Observations of misconduct | Problem |
|  | 2 | .88 | New ideas | Suggestion |
|  |  |  | His or her opinion regarding issues within the organization | Opinion |
|  |  |  | Advice that deviates from the norm | Opinion |
|  |  |  | Remarks that highlight problems in the organization | Problem |
|  | 3 | .85 | Plans to change procedures or processes | Suggestion |
|  |  |  | Spontaneous suggestions | Suggestion |
|  |  |  | Personal advice | Opinion |
|  |  |  | Attention towards inefficiencies in the workplace | Problem |
|  | 4 | .86 | Suggestions for improvement | Suggestion |
|  |  |  | His or her opinion, even if it is different from the view of others | Opinion |
|  |  |  | Concerns about processes that are not functioning optimally | Problem |

**Note.** One item ‘My colleague / subordinate offers technical tips’ was deleted from the 15- item quantity scale because it showed poor factor loadings and reduced reliability of the scale.

|  |  |  |  |  |
| --- | --- | --- | --- | --- |
| **TABLE D - All Voice Quality Items and Parcel Loadings of the 4 Factor- Model** | | | | |
| **Rater** | **Parcel** | **Loadings** | **Items** | **Category** |
|  |  |  | **Please indicate on a scale from 1 (completely disagree) to 7 (completely agree), to what extend you agree with the following statements regarding your colleagues/subordinates proactive and constructive comments** |  |
| Colleague | 1 | .91 | If my colleague shares an idea, it is original | Novelty |
|  |  |  | My colleague voices original advice |  |
|  |  |  | My colleague thinks about how his/her ideas should be implemented | Usefulness |
|  |  |  | When my colleague shares his/her concerns about dysfunctional processes, it is valuable |  |
|  | 2 | .82 | *My colleagues advice is hardly innovative.(CB) | Novelty |
|  |  |  | When my colleague offers me advice, it is usually refreshing. |  |
|  |  |  | If my colleague presents a new product or method, it is carefully thought through. | Usefulness |
|  |  |  | When someone else voices an idea, my colleague comes up with useful additions. |  |
|  | 3 | .74 | My colleague has a refreshing opinion. | Novelty |
|  |  |  | My colleagues’ opinion is original. |  |
|  |  |  | My colleagues’ ideas have high implementation value. | Usefulness |
|  |  |  | *In practice, my colleagues ideas do not work. (CB) |  |
|  | 4 | .91 | My colleagues' suggestions are original. | Novelty |
|  |  |  | If my colleague shares a suggestion, it is usually innovatory. |  |
|  |  |  | Advice my colleague offers is useful to me. | Usefulness |
| Supervisor | 1 | .89 | If my subordinate shares a suggestion, it is usually innovatory. | Novelty |
|  |  |  | *My subordinates advice is hardly innovative.(CB) |  |
|  |  |  | My subordinates ideas have high implementation value. | Usefulness |
|  |  |  | If my subordinate presents a new product or method, it is carefully worked out. |  |
|  | 2 | .95 | My subordinates opinion is original. | Novelty |
|  |  |  | When my subordinate offers me advice, it is usually refreshing. |  |
|  |  |  | My subordinate thinks about how his/her ideas should be implemented | Usefulness |
|  |  |  | Advice my subordinate offers is useful to me. |  |
|  | 3 | .93 | My subordinate has a refreshing opinion. | Novelty |
|  |  |  | My subordinate voices original advice. |  |
|  |  |  | *In practice, my subordinates ideas do not work. (CB) | Usefulness |
|  |  |  | When my subordinate shares his/her concerns about dysfunctional processes, it is valuable |  |
|  | 4 | .92 | If my subordinate shares an idea, it is original | Novelty |
|  |  |  | My subordinates' suggestions are original. |  |
|  |  |  | When someone else voices an idea, my subordinate comes up with useful additions. | Usefulness |
| All blocks of (randomized) quality items were preceded by control questions. Examples of control questions are: ‘The questions I will answer next are about a) the quantity of ideas, suggestions, opinions or advice my employee voices b) ‘The quality of … my employee voices’ c) The practical use of …..’ – or- ‘The questions I will answer next are about comments that are a) intended to constructively change and improve work processes in the organization, b) intended to ventilate ones’ personal opinion’. Only raters who were able to identify self-initiated constructive intent and the distinct aspects of quality and quantity were allowed to rate the quality and quantity scales. | | | | |
|  |  |  |  |  |
|  |  |  |  |  |
|  |  |  |  |  |
|  |  |  |  |  |

|  |  |  |  |  |  |  |  |  |  |  |  |
| --- | --- | --- | --- | --- | --- | --- | --- | --- | --- | --- | --- |
| **TABLE E (study 3). Descriptives and zero order correlations between, Cronbach’s alphas on the diagonal.** | | | | | | | | | | | |
|  |  |  |  |  | 1 | 2 | 3 | 4 | 5 | 6 | 7 |
|  | Variable | Rating | *Mean* | *SD* | **WMC** | **VQT** | **VQT Col** | **VQT Sup** | **VQL** | **VQL Col** | **VQL Sup** |
| 1 | **WMC** | Test | 5.26 | 1.18 | (.73) |  |  |  |  |  |  |
| 2 | **Voice Quantity** | Total | 3.18 | .55 | .13 | x |  |  |  |  |  |
| 3 |  | Colleagues | 3.2 | .63 | .10 | .80** | (.90) |  |  |  |  |
| 4 |  | Supervisors | 3.15 | .71 | .12 | .84** | .34** | (.91) |  |  |  |
| 5 | **Voice Quality** | Total | 4.35 | .78 | .24** | .71** | .48** | .68** | x |  |  |
| 6 |  | Colleagues | 4.36 | .84 | .18* | .52** | .48** | .38** | .76** | (.91) |  |
| 7 |  | Supervisors | 4.34 | 1.08 | .21* | .63** | .33** | .69** | .86** | .33** | (.95) |
| **Note.** * *p* < .05, ** *p* < .01, *** *p* < .001. | | | | |  |  |  |  |  |  |  |

| **TABLE F. Voice Quality and Quantity, Nomological Network** | | | | | | | | | | | | | | | | | | |
| --- | --- | --- | --- | --- | --- | --- | --- | --- | --- | --- | --- | --- | --- | --- | --- | --- | --- | --- |
| **Source** |  |  | **Manager** | | | | | |  | **Colleague** | | | | |  | **Self** | | |
|  |  |  |  | **Voice** | | | | Taking Charge |  | **Voice** | | | | Personal Initiative |  | Personal Initiative | Taking Charge | Proactive Person. |
|  |  | **Correlations** | Perform. | Quality | Quantity | Novel | Useful |  |  | Quality | Quantity | Novel. | Useful |  |  |  |  |  |
| **Manager** |  | Performance | (.83) |  |  |  |  |  |  |  |  |  |  |  |  |  |  |  |
|  |  | Voice Quality | .56^***^ | (.97) |  |  |  |  |  |  |  |  |  |  |  |  |  |  |
|  |  | Voice Quantity | .12 | .44^***^ | (.91) |  |  |  |  |  |  |  |  |  |  |  |  |  |
|  |  | Voice Novelty | .48^***^ | .95^***^ | .40^**^ | (.96) |  |  |  |  |  |  |  |  |  |  |  |  |
|  |  | Voice Usefulness | .59^***^ | .94^***^ | .42^**^ | .78^***^ | (.95) |  |  |  |  |  |  |  |  |  |  |  |
|  |  | Taking Charge | .42^***^ | .76^***^ | .51^**^ | .69^***^ | .75^**^ | (.94) |  |  |  |  |  |  |  |  |  |  |
| **Colleague** |  | Voice Quality | .18^*^ | .35^***^ | .25^**^ | .32^***^ | .35^**^ | .25^**^ |  | (.95) |  |  |  |  |  |  |  |  |
|  |  | Voice Quantity | .06 | .31^***^ | .40^**^ | .32^***^ | .26^**^ | .27^**^ |  | .38^***^ | (.88) |  |  |  |  |  |  |  |
|  |  | Voice Novelty | .12 | .32^***^ | .19^*^ | .31^***^ | .28^***^ | .20^*^ |  | .92^***^ | .40^***^ | (.92) |  |  |  |  |  |  |
|  |  | Voice Usefulness | .20^*^ | .32^***^ | .28^***^ | .26^**^ | .35^***^ | .27^**^ |  | .92^***^ | .30^***^ | .69^***^ | (.95) |  |  |  |  |  |
|  |  | Personal Initiative | .20^*^ | .40^***^ | .37^***^ | .33^***^ | .41^***^ | .32^***^ |  | .75^***^ | .42^**^ | .62^***^ | .76^**^ | (.87) |  |  |  |  |
| **Self** |  | Personal Initiative | .12 | .15 | .12 | .12 | .16^*^ | .08 |  | .23^**^ | .16^*^ | .26^**^ | .16^*^ | .31^**^ |  | (.77) |  |  |
|  |  | Taking Charge | .00 | .06 | .11 | .07 | .05 | .11 |  | .24^**^ | .22^**^ | .24^**^ | .20^*^ | .25^**^ |  | .49^***^ | (.88) |  |
|  |  | Proactive Personality | .04 | .13 | .01 | .15 | .10 | .05 |  | .18^*^ | .10 | .22^**^ | .11 | .19^*^ |  | .64^***^ | .56^***^ | (.84) |
| **Note.** * *p* < .05, ** *p* < .01, *** *p* < .001. This Zero-Order correlation table is based on a scale-development Field study of 168 employees and their colleagues and managers. All items in the voice quantity / quality scales are also present in the measures of Study 3. Performance items are the managers evaluation of the employee on his / her last performance evaluation on the following dimensions: (1) quality of work, (2) achieving work goals, (3) ability to maintain social relations, (4) ability to work in a team-context, (5) ability to get work done in a timely fashion. Other proactive concept scales are derived from validated studies on personal initiative (Frese, Kring, Soose, & Zempel, 1997) , taking charge (Morrison & Phelps, 1999) and proactive personality (Bateman & Crant, 1993). | | | | | | | | | | | | | | | | | | |
|  |  |  |  |  |  |  |  |  |  |  |  |  |  |  |  |  |  |  |
|  |  |  |  |  |  |  |  |  |  |  |  |  |  |  |  |  |  |  |
|  |  |  |  |  |  |  |  |  |  |  |  |  |  |  |  |  |  |  |

**REFERENCES**

Bagozzi, R. P., & Edwards, J. R. (1998). A General Approach for Representing Constructs in Organizational Research. *Organizational Research Methods*, *1*(1), 45–87.

Nasser-Abu Alhija, F., & Wisenbaker, J. (2006). A Monte Carlo Study Investigating the Impact of Item Parceling Strategies on Parameter Estimates and Their Standard Errors in CFA. *Structural Equation Modeling: A Multidisciplinary Journal*, *13*(2), 204–228.

Van Dyne, L., & LePine, J. A. (1998). Helping and Voice Extra-Role Behaviors: Evidence of

Construct and Predictive Validity. *The Academy of Management Journal*, *41*(1), 108–119

1. We also fitted a 4-factor model with Type (Voice Quality vs. Quantity) as the higher order construct, which resulted in an equally acceptable fit (*χ²* = 195.94, *df* = 101, *N*=144, *p* < .001, CFI = 0.95, TLI 0.95, RMSEA =.08, 90% CI [ .06 , .09 ], AIC = 4084, BIC = 4235). This model is was also significantly better than the 2-factor source model (*Δχ²* = 368.66*, df =2* , *p* < .001). [↑](#footnote-ref-1)
